# Preterm Labor: Understanding of the Mechanism Involved to Improve Prediction and Prevention

**DOI:** 10.1155/2013/858506

**Published:** 2013-07-24

**Authors:** S. E. Illanes, J. K. Nien, G. E. Rice

**Affiliations:** ^1^Department of Obstetrics and Gynecology and Laboratory of Reproductive Biology, Universidad de Los Andes, Las Condes, 7620001 Santiago, Chile; ^2^The University of Queensland Centre for Clinical Research, RBWH Campus, Herston, Brisbane, QLD 4029, Australia

Preterm birth is a significant problem occurring in around 10% of all deliveries and its increase is remarkable, even in developed countries. Being born under 37 weeks, and most importantly under 32 weeks, is the major cause of perinatal morbidity and mortality and accounts for the majority of neonatal deaths. Recent advances in perinatology and neonatology have increased survival rates, particularly for the extremely preterm baby. The associated morbidity for these survivors' remains significant, and up to one-quarter will have at least one major disability. This special issue focused in some aspect of the physiopathology of the preterm labor and different strategies to predict the patient at risk in order to improve the prevention of this pathology.

This issue highlights the diversity of the physiopathology of preterm labor by including different points of view, such as focusing in the expression of steroid receptor coactivators in fetal membranes and its relationship with preterm labor. Using immunohistochemistry, western blot analysis, and real-time PCR, they demonstrate that SRC-1, SCR-2, p300, and PCAF proteins are present in nuclear extracts and display tissue-specific and labor-associated differential expression. 

In another paper, the authors propose a novel putative target for the prevention of infection-triggered preterm labor. They suggest that uterotonic effects of endothelin-1 (E-1) are mediated via sphingosine kinase (SphK). E-1 is a molecule that exerts potent oxytocic effects and is elevated in amniotic fluid obtained from preterm births. SphK plays a key role in ET-1-induced myometrial contractions and may represent a potential target for tocolysis to prevent preterm birth. 

In terms of prediction, a review accessing the accuracy of transvaginal sonographic cervical length in predicting preterm birth in women with twin pregnancy and symptoms of preterm birth is presented. The authors present a meta-analysis, using bivariate regression analysis, accounting for correlation between sensitivity and specificity. Despite the large variation in definition of preterm birth and cut-off point of cervical length and gestational age, they concluded that there is insufficient evidence to recommend the measurement of cervical length as a predictor of preterm birth in women with a twin pregnancy and symptoms of preterm birth because specificity and sensibility are low.

With respect to prevention of preterm birth, a systematic review of the use of cervical pessaries and adjunctive therapies to cerclage is presented. The aim of one study was to evaluate the effective use of the cervical pessary to prevent preterm birth before 28, 34, and 37 weeks of gestation. Interestingly, one randomized controlled trial demonstrated a lower delivery rate prior to 34 weeks in the pessary group. Other studies, however, showed no positive effect. The author concluded that the cervical pessaries represent an affordable, safe, and reliable alternative for prevention of preterm birth in selected at-risk pregnant women. Nevertheless, more randomized clinical trials are needed before this device can be used in clinical practice. In another study, the authors reviewed publications that assessed the efficacy of adjunctive therapies used in addition to cervical cerclage as a preventive measure for preterm birth. The adjunctive therapies identified were progesterone, reinforcing or second cerclage placement, tocolytics, antibiotics, bedrest, and pessary. Even though small numbers of patients limited the strength of the conclusions, none of the studies clearly show the benefit of any adjunctive therapy used.

By compiling these papers, we hope to improve the knowledge of clinicians and researchers with respect to this important cause of perinatal morbidity and mortality. Increasing the understanding of the physiopathology of the preterm labor and the strategies to prevent it are essential steps to avoid the consequence of this disease.

*S. E. Illanes* *S. E. Illanes* *J. K. Nien* *J. K. Nien* *G. E. Rice* *G. E. Rice*

